# The safety and effectiveness of a modified guidewire pigtailing technique in transesophageal echocardiography-guided percutaneous closure of secundum atrial septal defects

**DOI:** 10.1186/s12872-026-05576-4

**Published:** 2026-02-03

**Authors:** Huipin Hua, Peng Miao, Xun Zhang, Shucan Xu, Jing Jin, Jun Shao

**Affiliations:** 1 Department of Cardiovascular Surgery, The Second Affiliated Hospital of Nanjing Medical University, Nanjing, 210011 P. R. China; 2 Department of Cardiac Ultrasound, The Second Affiliated Hospital of Nanjing Medical University, Nanjing, 210011 P. R. China; 3 Department of Cardiology, Binhai People’s Hospital, Yancheng, 224500 P. R. China

**Keywords:** Atrial septal defect, Percutaneous closure, Transesophageal echocardiography, Wire pigtailing

## Abstract

**Background:**

To evaluate the safety and effectiveness of a guidewire pigtailing technique in percutaneous atrial septal defect(ASD) closure guided by transesophageal echocardiography guidance.

**Methods:**

We retrospectively collected and analyzed clinical data of 137 patients who underwent transesophageal echocardiography(TEE) guided percutaneous secundum atrial septal defect closure from September 2022 to December 2024 at the Department of Cardiovascular Surgery in the Second Affiliated Hospital of Nanjing Medical University. Patients were divided into the study groups (guidewire pigtailing group, with the guidewire tip looped within the left atrium, *n* = 78) and the control group (with the guidewire tip positioned in the left superior pulmonary vein, *n* = 59) based on the surgical technique used. The technique’s success rate, operation time, postoperative new-onset arrhythmia, pericardial effusion, residual shunt, and vascular access-related complications were compared between the two groups.

**Results:**

Gender, age, atrial septal defect diameter, follow-up time, and operation time between the two groups were comparable ( *P* > 0.05 ). Surgical success was achieved in all 137 patients. Within the study group, four cases developed new-onset postoperative arrhythmias, including two cases of premature atrial contractions(PACs) and two cases of incomplete right bundle branch block(IRBBB). In the control group, one patient was converted to the pigtailing technique due to difficulty advancing the guidewire into the left superior pulmonary vein(LSPV), and one case developed new-onset pericardial effusion, which decreased after one week of observation and resolved completely at three-month follow-up; one case experienced a puncture site hematoma, which improved with compression bandaging; and three cases developed new-onset postoperative arrhythmias, all diagnosed as IRBBB. The two groups of patients completed 3 ~ 15 months of follow-up. Follow-up data were comparable between groups, with no residual shunt, occluder dislodgement, and access site related complications.

**Conclusion:**

The guidewire pigtailing technique for percutaneous secundum atrial septal defect closure under transesophageal echocardiography guidance was found to be a safe and feasible procedure, which may help reduce the learning curve and procedural complexity, and serves as a valuable alternative to the standard LSPV access.

**Supplementary Information:**

The online version contains supplementary material available at 10.1186/s12872-026-05576-4.

## Background

 Atrial septal defect (ASD), one of the most frequent forms of congenital heart disease, accounts for approximately 30% of such cases [1]. Current evidence [2] attributes cardiac malformations and heart failure primarily to congenital developmental anomalies and genetic factors. Without intervention, ASD can lead to complications such as pulmonary arterial hypertension (PAH), as well as stroke and ischemic heart disease [3, 4]. Presently, percutaneous ASD closure is the preferred treatment for secundum-type ASD [5], owing to its advantages of reduced short- and long-term mortality and lower complication rates [6, 7]. Conventional procedures necessitate digital subtraction angiography (DSA), exposing both clinicians and patients to ionizing radiation [8]. In recent years, ultrasound-guided ASD closure has gained increasing favor as an alternative [9]. Two modalities exist in ultrasound-guided ASD closure: transthoracic echocardiography (TTE) and transesophageal echocardiography (TEE). TEE provides superior visualization of the atrial septal structure and adjacent tissues, particularly in cases involving small defects or complex anatomy [10], and demonstrates safety in guiding interventions for ASD [11]. Nevertheless, this measure has not been widely adopted as it failed to demonstrate superiority over fluoroscopy-guided closure. Traditionally, in TEE-guided ASD closure, the guidewire has to be delivered to the left superior pulmonary vein [9, 12], and this step minimizes the possibility of iatrogenic injury and pericardial effusion upon entry of the delivery system and during closure device deployment [12]. However, the passage of the guidewires into the left superior pulmonary vein is often time-consuming and can result in complications, as complete TEE guidance often fails to intuitively guide wires into the left superior pulmonary vein. And it is often technically challenging and time-consuming, with a long learning curve [13]. To overcome this technical dilemma, we adopted a guidewire pigtailing technique that enhances visualization while minimizing the complexity of the procedure. In this study, we conducted a retrospective cohort study including ultrasound-guided ASD closure patients treated at our center and determined the efficacy and safety of this new technique.

## Methods

### Study design and patient population (Retrospective Analysis)

This was a retrospective study that included 137 ASD patients who were treated with ultrasound-guided percutaneous closure at our institution between September 2022 and December 2024. Patients were divided into the study groups (guidewire pigtailing group, with the guidewire tip looped within the left atrium, *n* = 78) and the control group (with the guidewire tip positioned in the left superior pulmonary vein, *n* = 59). The disparity in sample sizes between the two groups (*n* = 78 vs. *n* = 59) reflects a temporal shift in our center’s clinical practice. As the guidewire pigtailing technique demonstrated ease of use and consistent safety during the initial implementation, it was increasingly adopted by operators as the preferred strategy during the latter phase of the study period.

*Inclusion Criteria* [14]: (1) Patients aged 2 years or older, or with a body weight of 10 kg or greater; (2) Secundum ASD without other defects or evidence of right heart volume overload; (3) sufficient rims: at least 5 mm from the coronary sinus, superior vena cava, inferior vena cava, or pulmonary veins, and 7 mm from the atrioventricular valves.

*Exclusion criteria*: (1) primum or sinus venosus ASD; (2) active endocarditis; (3) emboli at the puncture site or within the ASD; (4) pulmonary arterial hypertension with right-to-left shunting; (5) coexisting of severe myocardial or valvular diseases unrelated to ASD(including dilated or hypertrophic cardiomyopathy, significant ventricular dysfunction (LVEF < 40%), or clinically significant valvular heart disease (moderate-to-severe stenosis or regurgitation)); (6) History of systemic infectious disease within the preceding month or uncontrolled active infections; (7) presence of thrombi in the left atrium or left atrial appendage, accompanied by localized or generalized pulmonary venous return anomalies, left atrial septation, or hypoplasia of the left atrium or left ventricle; (8) allergy to the occluder material (nickel-titanium alloy); (9) co-existence of other cardiac anomalies.

To ensure the comparability of the two groups and minimize confounding factors, strict anatomical and clinical screening was performed. All included patients possessed sufficient rims for percutaneous closure, and those with severe comorbidities were excluded, ensuring a homogeneous baseline across both cohorts.

### Instruments and surgical methods

**Fig. 1:**
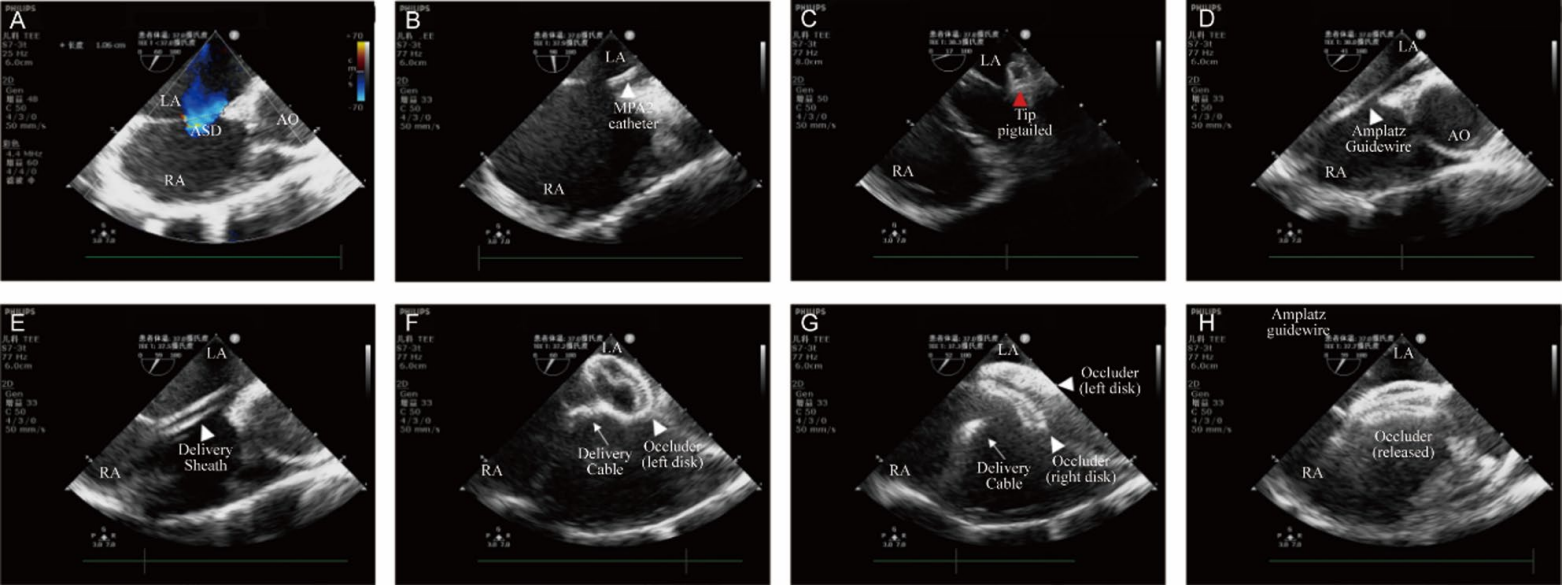
Transesophageal Echocardiography (TEE)-Guided Percutaneous ASD Closure Procedure. **A** Pre-procedural TEE assessment of the ASD diameter and rims. **B **The MPA2 catheter is advanced through the defect into the left atrium (LA). **C** The Amplatz super-stiff guidewire is advanced, with its tip curling into a “pigtail” shape (red arrowhead) within the LA to prevent wall injury. **D** The stiffened guidewire traverses the ASD, establishing a stable rail. **E** The delivery sheath is advanced over the wire; the parallel hyperechoic walls of the sheath and the inner wire create the characteristic “double-track sign” (white arrowhead), confirming correct trans-septal positioning. **F** Deployment of the left atrial disc. **G** Deployment of the right atrial disc. **H** Final release of the occluder after confirming stability. LA: Left Atrium; RA: Right Atrium; Ao: Aorta

A Philips CX50 color Doppler ultrasound system was used for intraoperative TEE imaging. Following general anesthesia, a transesophageal ultrasound probe was inserted into the esophagus to evaluate the size and position of the atrial septal defect across multiple imaging planes. Specifically, the mid-esophageal bicaval view (90°–110°) was used to assess the superior and inferior rims and guide the catheter path, while the short-axis view (30°–60°) was utilized to evaluate the aortic rim. This approach facilitates precise measurement of the distances between defect margins and adjacent structures, enables determination of eligibility for closure, and selection of an appropriately sized occluder device based on the measured ASD dimensions (see Fig. 1A). All procedures were conducted in a hybrid operating suite, allowing for immediate conversion to X-ray-guided closure or open-heart surgical repair in the event of unforeseen complications.

*For both groups*, percutaneous access was established via ultrasound-guided right femoral vein puncture. Following insertion of the vascular sheath (SCW Medicath, SCW-IS-0511), the working catheter distance is measured and marked using the distance from the puncture site to the 2nd intercostal space. Guided by TEE, a Terumo guidewire (RF*GA35153M ) with an MPA2 catheter (Cordis,534-542T) was maneuvered through the atrial septal defect into the left atrium (Fig. 1B).

**Fig. 2:**
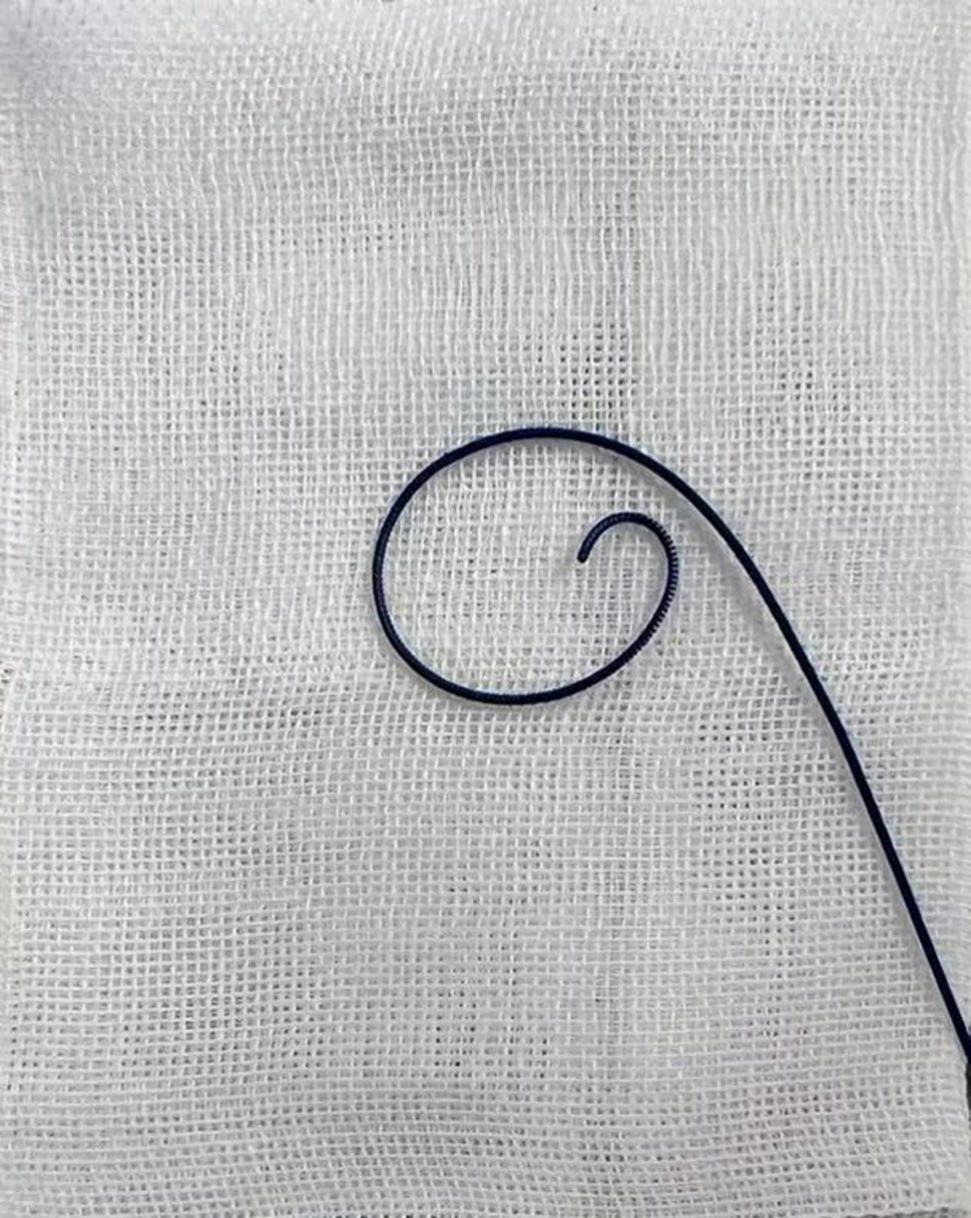
Wire pigtailing of the Amplatz Guidewire

*In the study group (wire pigtailing technique)*, a 0.35 mm*260 mm Amplatz super-stiff wire (Boston Scientific, M001465020) is used. Before insertion, the distal soft tip of the guidewire is manually shaped into a ‘pigtail’ curve (Fig. 2). Specifically, the operator gently applies traction to the flexible tip; due to the guidewire’s inherent elasticity, the tip curls naturally into a loop upon release. By modulating the traction, a loop diameter of approximately 1.5–2.0 cm is consistently achieved. This specific diameter ensures clear visualization under TEE without occupying excessive atrial volume. Since the loop is formed by the wire’s elastic recoil rather than rigid deformation, it maintains excellent shape stability within the left atrium. All operators followed this standardized protocol to ensure technical consistency. Over various planes, TEE first ensures that the tip of the catheter remains within the central area of the left atrium, not against the left atrium wall, within or at the opening of the left atrial appendage. The guidewire is removed and subsequently exchanged for the pigtailed Amplatz super-stiff wire while holding the catheter in place. While the Amplatz super-stiff wire approaches the catheter tip, wire sliding within the catheter can be observed. The catheter and subsequently the introducer sheath are slowly retracted while the stiff wire is held in place, exposing the pigtail in the left atrium (Fig. 1C).

*In the control group (left superior pulmonary vein technique)*, the Amplatz 0.35 mm*260 mm Amplatz super-stiff wire is left intact. Our approach is similar to that described by Pan et al., following entry into the left atrium, the catheter and guide wire are advanced into the left superior pulmonary vein. The catheter is held in place, and the guidewire is exchanged for the super-stiff wire. After confirmation that the stiff wire holds its place in the left superior pulmonary vein, the catheter and introducer sheath are removed.

In both groups, an appropriately sized delivery sheath that matches the desired occluder is introduced and advanced along the guidewire (Fig. 1D). As the dilator tip passes through the ASD and advances, switching between different TEE planes is needed to prevent it from advancing too deeply. The dilator is subsequently withdrawn, and at this stage, the double rail sign can be visualized (Fig. 1E). At this stage, the inner dilator is removed together with the stiff wire, and the occluder attached to a delivery cable is pushed forward until it reaches the tip of the delivery sheath. At this stage, the sheath is retracted while holding the delivery cable still to first release the occluder disk on the left atrial side (Fig. 1F). The delivery cable is pulled backwards, and the occluder is anchored to the septum in the left atrium, following which the sheath is pulled back again to release the right disk. Repeated push-pull tests are performed under TEE to assess for secure anchorage (Fig. 1G). Before and after releasing the occluder, the absence of interference with the atrioventricular valves, superior or inferior vena cava, coronary sinus, and residual shunt is confirmed (Fig. 1H). All catheters and sheaths were subsequently removed, and the puncture site was manually compressed before being bandaged.

### Follow-up

Post-discharge follow-up was conducted via outpatient visits at 3 months, 6 months, and 1 year post-procedure. Evaluations included transthoracic echocardiography, electrocardiography, and chest X-ray imaging. These assessments monitored the position and morphology of the occluder device, residual shunting, pericardial effusion, and the incidence of arrhythmias.

### Statistical analysis methods

Statistical Analysis Methods Data analysis was performed using SPSS version 26.0 statistical software (IBM Corp, Armonk, NY). Continuous variables were first assessed for normality using the Shapiro-Wilk test and for homogeneity of variance using Levene’s test. Normally distributed data are expressed as means ± standard deviations. Intergroup comparisons were performed using the independent samples t-test; however, for variables demonstrating heteroscedasticity (unequal variances, e.g., operative time), Welch’s t-test was adopted to correct for the disparity. Categorical variables were reported as counts and percentages. Intergroup differences were assessed using the chi-square test; Fisher’s exact test was used when the theoretical frequency in any cell was less than 5 (e.g., low-incidence complications). A P-value < 0.05 was considered indicative of statistical significance.

## Results

Baseline clinical characteristics of both groups are presented in Table 1.

All patients in both the study (wire pigtailing technique) and control (left superior pulmonary vein technique) groups successfully completed the ASD closure procedures. Baseline characteristics, including sex, age, body mass index, and ASD diameter, are comparable between groups.

The surgical success rate (defined as successful device implantation and defect closure) was 100% in both groups (Table 3). However, the technical success rate (defined as successful closure using the initially assigned guidewire strategy) was 100% in the study group and 98.3% in the control group (Table 3). The discrepancy in the control group was due to one patient in whom the LSPV access failed due to anatomical difficulties; the procedure was successfully completed by converting to the guidewire pigtailing technique. The average total surgery time, defined as the time interval from successful puncture to delivery sheath removal, was comparable in both groups (5.0 ± 0.8 min vs. 5.5 ± 2.6 min). Statistical analysis showed no significant difference between the two groups (*P* = 0.115), indicating that the pigtailing technique achieved comparable procedural efficiency to the conventional method. In terms of post-surgical complications, paroxysmal arrhythmias occurred in four patients (5.1%) in the study group (comprising 2 cases of premature atrial contractions [PACs] and 2 cases of incomplete right bundle branch block [IRBBB]) and three patients (5.1%) in the control group (all 3 cases were IRBBB); all episodes spontaneously resolved under monitoring. One patient in the control group had mild pericardial effusion without symptoms, which was stable throughout the hospital stay and discharge. One patient within the control group had a small puncture site hematoma during the hospital stay. No other post-procedural complications were observed. Post-Procedure Complications of both groups are presented in Table 2.

The follow-up time in both groups was similar (Table 3). The median follow-up duration was 6 months (range: 3–15 months) for both groups. Specifically, 31 patients in the guidewire pigtailing group and 26 patients in the control group completed the one-year follow-up. At one-year follow-up, no residual shunting, device dislodgement, or evidence of obstruction in valvular or vena cava flow was noted. There were no delayed peripheral vascular injuries reported in either group.

**Table 1:** Baseline clinical characteristics of patients

Male, *n* (%)	Wire pigtailing Group (*n* = 78)	Left superior pulmonary vein Group (*n* = 59)	T/χ²	*P*
	34(43.6)	21(35.6)	0.894	0.344
Age (years), mean ± SD	13.2 ± 10.3	12.9 ± 11.6	3.791	0.054
Weight (kg), mean ± SD	36.1 ± 15.1	34.3 ± 16.8	1.357	0.246
Height (m), mean ± SD	1.40 ± 0.22	1.34 ± 0.26	2.189	0.141
BMI (kg/m²), mean ± SD	17.6 ± 3.4	18.0 ± 4.0	3.595	0.060
ASD Diameter (mm), mean ± SD	8.0 ± 4.8	8.2 ± 5.9	0.760	0.385
Sufficient Rims, n (%)	78 (100%)	59 (100%)	-	> 0.99 *
Major Comorbidities, n (%)	0 (0%)	0 (0%)	-	> 0.99 *

Values are presented as mean ± standard deviation (SD) or number (percentage). *LSPV* Left Superior Pulmonary Vein, *BMI* Body Mass Index, *ASD* Atrial Septal Defect. * Comparison performed using Fisher’s exact test. Sufficient rims were defined as >5 mm from critical structures

**Table 2:** Post-Procedure complications data

Total Post-Procedure Complications	Wire pigtailing Group (*n* = 78)	Left superior pulmonary vein Group (*n* = 59)	*P* *
	4(5.1)	5(8.5)	0.434
Premature atrial contractions	2(2.6)	0	0.505
Incomplete right bundle branch block	2(2.6)	3(5.1)	0.390
Pericardial Effusion	0	1(1.7)	0.431
Puncture site hematoma	0	1(1.7)	0.431

Values are presented as number (percentage).* *P* values were calculated using Fisher’s exact test due to the low frequency of events in the contingency tables

**Table 3:** Surgical and Follow-Up data

Operative Time (min), mean ± SD	Wire pigtailing Group (*n* = 78)	Left superior pulmonary vein Group (*n* = 59)	T/χ²	*P*
	5.0 ± 0.8	5.5 ± 2.6	-1.60	0.115 ^a^
Surgical success(%)	78(100)	59(100)	-	-
Technical success(%)	78(100)	58(98.3)	-	> 0.99 ^b^
Follow-Up Duration (months), median	6	6	-0.706	0.480

Technical success refers to the successful completion of the procedure using the initially assigned guidewire technique. Surgical success refers to the ultimate successful closure of the ASD. ^a^ *P* value calculated using Welch’s t-test to account for unequal variances (heteroscedasticity) between groups. ^b^ Calculated using Fisher’s exact test

## Discussion

King and colleagues successfully employed a double-umbrella device for catheter-based ASD closure in 1975; percutaneous ASD closure has emerged as the preferred therapeutic approach. The vast majority of ASDs can be effectively treated with this technique [15], which is not only favored by the patients but is also effective in reducing hospital stay and total health expenditure [16]. Conventional procedures rely on DSA, necessitating exposure of both clinicians and patients to ionizing radiation for prolonged periods. While these procedures typically do not require prolonged X-ray exposure, studies have demonstrated a strong association between even low-dose radiation exposure and the incidence of cancers, such as leukemia [17]. Wen et al. [18] first reported the successful ultrasound-guided closure of ASDs without fluoroscopy in 19 patients in China in 1999. Over the years, echocardiography-guided ASD closure has proven safe and reliable [19–21].

An important step in the TEE-guided ASD closure is LSPV access [22], which provides better coaxiality support while minimizing the risk of injury to the atrium and its surrounding structures. However, in some patients, this can be challenging. TEE often provides a two-dimensional guidance for the operator, and frequent attempts to reach LSPV can succeed at the expense of increased incidence of iatrogenic surrounding structure injury, particularly the left atrial appendage. While ICE can overcome some of the challenges, it is often more expensive, particularly in China. Here, we described a simple yet safe technique to overcome the difficulties encountered when accessing the LSPV.

Compared to the standard LSPV technique, our pigtail technique can be achieved at no extra cost. Pigtailing of the Amplatz guidewire provides several benefits. It provides a better signal through TEE. A straight guidewire may be prone to artifacts and noise that interfere with the signal on TEE. Access to the LSPV can be particularly challenging in cases of unwanted artifacts on the path or at the ostium of LSPV. Another benefit is that the pigtailing helps protect the left atrium from trauma and perforation, which may be detrimental in a minimally invasive procedure like ASD closure. Our study observed a difference in complication profiles that warrants discussion. In the control group, one patient developed pericardial effusion. This is a known risk of the conventional LSPV technique, where the stiff guidewire, if not perfectly coaxial or if manipulated blindly due to poor ultrasound visualization, can exert excessive point pressure on the thin left atrial wall or appendage, potentially leading to micro-perforation or injury. In contrast, the guidewire pigtailing technique theoretically mitigates this risk through a mechanical advantage: manually shaping the tip into a loop increases the contact surface area between the wire and the atrial wall. This effectively converts high-risk ‘point pressure’ into safer ‘distributed surface pressure,’ minimizing the risk of perforation even if the wire contacts the atrial roof or appendage wall. This mechanism likely explains the absence of pericardial effusion in the study group. Although puncture site hematoma (observed in one control patient) is primarily related to venous access, the simplified maneuvering of the pigtailing technique may reduce overall procedural time and catheter manipulation, potentially contributing to general safety. Importantly, pigtailing also helps the wire to anchor within the left atrium chamber, a step that is critical for the advancement of the delivery system.

Ideally, stiff wires are preferred in ASD closure to provide a stable ‘rail’ for advancing the large-caliber delivery sheath. Traditional flexible wires, such as the Inoue wire, are inadequate for this purpose primarily due to system incompatibility; they are typically designed for a 0.025-inch platform, whereas the delivery system used in our study requires a 0.035-inch platform. Furthermore, flexible wires lack sufficient mechanical stiffness. When advancing a rigid delivery sheath over a flexible wire (particularly a smaller caliber 0.025-inch wire), the assembly is prone to prolapsing back into the right atrium due to inadequate support. Therefore, our technique utilizes the 0.035-inch Amplatz super-stiff wire to ensure robust support, while the pigtailing modification converts the traumatic tip into a safe, visible loop. This combination effectively mitigates perforation risks and simplifies the procedure, thereby helping to reduce the learning curve for TEE-guided closure.

Our data also supports the above notions. The pigtailing group also demonstrated a favorable technical success with a safety profile comparable to that of the conventional technique. It is worth noting that this technique can also serve as a backup in challenging LSPV access cases for those who still prefer a traditional TEE-guided closure. In fact, we adopted this technique in one patient within the control group due to difficult LSPV. Our comparable perioperative complication rate has also demonstrated that this technique maintains a comparable safety profile.

Ultrasound-guided ASD closure can be categorized based on the imaging modality employed, encompassing TTE, intracardiac echocardiography (ICE), and TEE. TTE offers the advantages of procedural simplicity and non-invasiveness. However, its imaging clarity is often compromised in patients with thicker chest walls or emphysema. This limitation can result in reduced procedural precision and may potentially elevate the risk of unforeseen complications during closure. Furthermore, TTE exhibits limitations in evaluating the margins of ASD defects and certain forms of anomalous pulmonary venous drainage [23]. ICE is an effective modality for intraprocedural imaging, eliminating the need for deep sedation or general anesthesia and allowing operators full control over image acquisition. However, its use requires substantial expertise in catheter manipulation and image interpretation, and the high cost limits its accessibility, contributing to a long learning curve and constraining its broader adoption [24, 25]. In contrast, TEE involves positioning an ultrasound probe in the esophagus or stomach, enabling visualization from posterior or posteroinferior perspectives relative to the heart, rendering it particularly well-suited for guiding percutaneous ASD closure. TEE’s advantages include its independence from patient positioning, pulmonary shadowing, or chest wall thickness [10], facilitating precise assessment of the ASD’s location and its relationship with adjacent structures.

TEE typically necessitates general anesthesia, which carries a potential risk of esophageal injury [26]. However, in our clinical practice, no serious related complications were observed, potentially attributable to the use of laryngeal mask airways during general anesthesia. Furthermore, as the majority of patients with ASD at our center are adolescents and children, general anesthesia mitigates complications arising from patient movement. Multicenter studies have corroborated the safety of general anesthesia in the context of ASD closure procedures [9, 27]. And during TEE-guided procedures, the ultrasonographer’s imaging operations were performed concurrently with the surgeon’s puncture and related maneuvers, without significantly prolonging operative time.

Despite the demonstrated safety and efficacy of ultrasound-guided ASD closure, this technique has not yet achieved widespread clinical adoption, primarily due to its technical complexity. To address this challenge, Pan Xiangbin developed a novel guidewire system for interventional procedures. This guidewire features a spindle-shaped tip designed to enhance visibility under ultrasound while minimizing injury to critical structures, such as the left atrial posterior wall, left atrial appendage, and mitral valve [28]. However, this guidewire system has not yet been implemented in clinical practice.

In our clinical practice, we observed that the looped tip of the Amplatz guidewire produces a prominent, shadow-like image within the atrium, rendering it readily identifiable. Consequently, we employed the Amplatz guidewire pigtailing technique, whereby the stiffened guidewire’s tip is curved into a loop, providing sufficient support within the left atrium without compromising atrial structures and ensuring clear visualization across various positions. Furthermore, a comparative analysis of imaging under TTE and TEE revealed that TEE consistently captured all looped guidewires, whereas TTE yielded unclear visualization, likely attributable to the positional relationship between the ultrasound probe and the looped tip. This finding underscores one of the rationales for utilizing TEE to guide ASD closure. While our clinical outcomes are comparable to those achieved with the novel spindle-shaped guidewire system developed by Pan et al., the clinical applicability differs. Pan’s system is a specialized device designed specifically for ultrasound visibility, offering standardized industrial quality. In contrast, our pigtailing technique is a manual modification of a standard guidewire. The primary advantage of our approach is its universal accessibility and zero additional cost. It does not require regulatory approval for new device importation or additional hospital procurement, making it an immediately applicable solution for interventional cardiologists, especially in resource-limited settings.

Our findings demonstrate a 100% success rate for ASD closure under TEE guidance using the guidewire pigtailing technique, with no instances of occluder device replacement. The high success rate of the guidewire pigtailing technique may be primarily attributable to enhanced guidewire stability and reduced displacement.

Several limitations should be acknowledged. First, this is a non-randomized, retrospective study. The allocation of patients was influenced by the operator’s evolving preference. Although strict inclusion criteria ensured comparable baseline anatomy (sufficient rims) and absence of comorbidities between groups, this design inherently introduces selection bias. Second, the sample size (*n* = 137) is relatively small. This limits the statistical power of the study, meaning it may lack the sensitivity to detect significant differences in low-incidence complications between the two techniques. Therefore, the “comparable safety” observed should be interpreted with caution. Future research should prioritize multicenter, randomized controlled trials (RCTs) with larger cohorts to definitively validate these findings and eliminate potential biases.

Finally, the follow-up period was relatively short (median 6 months, range 3–15 months). While the primary advantage of the guidewire pigtailing technique lies in the perioperative phase—specifically in enhancing safety and visualization during deployment—we theoretically expect long-term outcomes to be comparable to standard techniques since the implanted device is identical. However, we acknowledge that current data are insufficient to definitively validate this expectation regarding late complications such as device erosion or arrhythmias. Therefore, continuous long-term follow-up is ongoing to provide definitive conclusions.

## Conclusion

TEE-guided percutaneous ASD closure represents a straightforward, safe, and minimally invasive surgical approach. Nevertheless, it entails a notable learning curve and demands a high level of technical proficiency. In our current series, the guidewire pigtailing technique was found to be a safe and feasible procedure. It offers operational simplicity, requires no additional consumables, and demonstrates a robust safety profile. Under ultrasound guidance, the looped tip is readily visualized, significantly reducing procedural complexity. Furthermore, this technique can serve as a valuable rescue strategy when conventional left superior pulmonary vein (LSPV) access is difficult or fails. While ultrasound-guided percutaneous ASD closure has progressively matured, randomized controlled trials or multicenter prospective studies are required for definitive conclusions. Additionally, long-term follow-up data require ongoing scrutiny to ensure sustained clinical outcomes.

## Supplementary Information

Supplementary Material 1.

Supplementary Material 2.

Supplementary Material 3.

## Data Availability

The datasets generated and/or analyzed during the current study are not publicly available due to patient privacy regulations but are available from the corresponding author, Jun Shao (Email: Shaojun1979nj@163.com), on reasonable request for non-commercial research purposes.

## Abbreviations

ASD: Atrial Septal Defect
TEE: Transesophageal Echocardiography
TTE: Transthoracic Echocardiography
LSPV: Left Superior Pulmonary Vein
ICE: Intracardiac Echocardiography
DSA: Digital Subtraction Angiography
LVEF: Left Ventricular Ejection Fraction
BMI: Body Mass Index
PAH: Pulmonary Arterial Hypertension
PACs: Premature Atrial Contractions
IRBBB: Incomplete Right Bundle Branch Block
RCT: Randomized Controlled Trial
