# Supplementary figures and images for: The safety and effectiveness of a modified guidewire pigtailing technique in transesophageal echocardiography-guided percutaneous closure of secundum atrial septal defects
Source: BMC Cardiovasc Disord. 2026 Feb 3;26:195. doi: 10.1186/s12872-026-05576-4 (PMC12958641; doi:10.1186/s12872-026-05576-4)

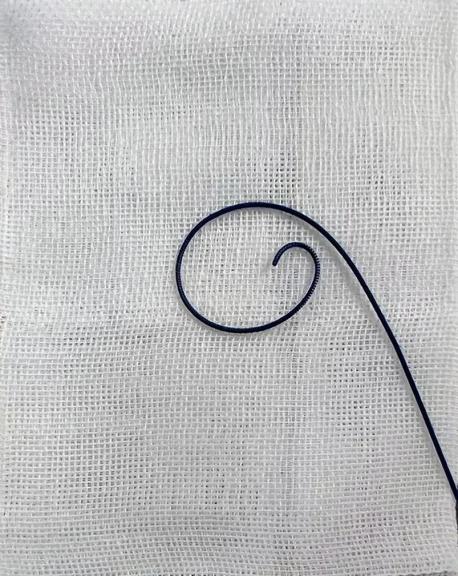

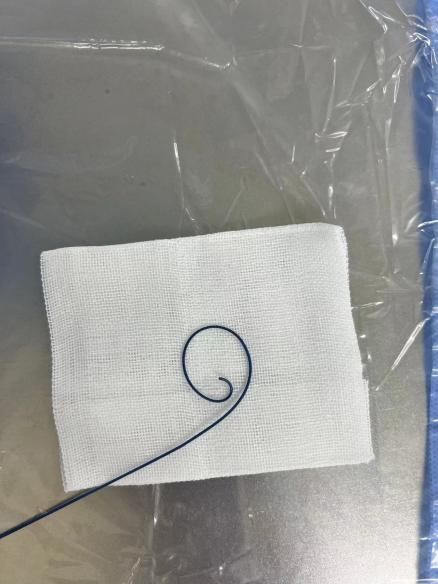

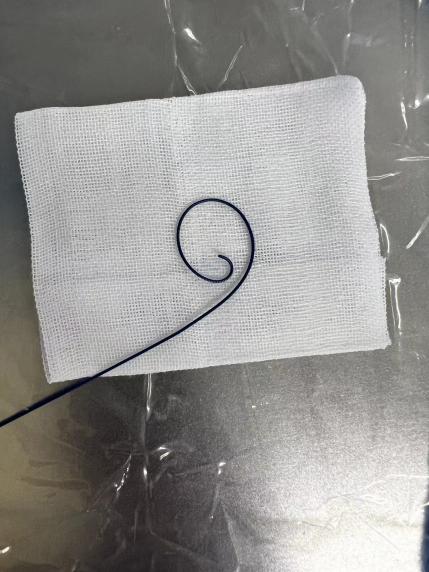


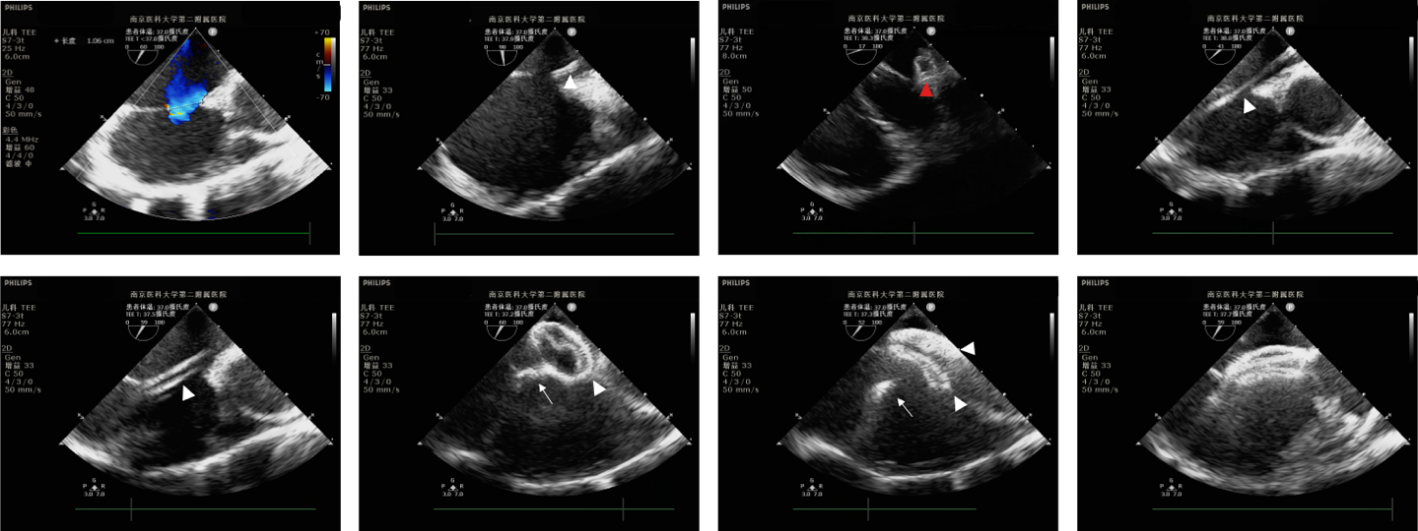

Supplement: Supplementary file 2 — Supplementary Material 2. [file 12872_2026_5576_MOESM2_ESM.docx]
