# Supplementary material for: The safety and effectiveness of a modified guidewire pigtailing technique in transesophageal echocardiography-guided percutaneous closure of secundum atrial septal defects
Source: BMC Cardiovasc Disord. 2026 Feb 3;26:195. doi: 10.1186/s12872-026-05576-4 (PMC12958641; doi:10.1186/s12872-026-05576-4)
Supplement: Supplementary file 3 — Supplementary Material 3. [file 12872_2026_5576_MOESM3_ESM.pdf]

# 科研伦理审查批件/意见

编号　2024-KY-019-01

| | | | |
|---|---|---|---|
| 项目名称 | 导丝成袢法在单纯食管超声引导下经皮房间隔缺损封堵术中的应用 — 单中心回顾性队列研究 | | |
| 申请人 | 邵峻 | 申请科室 | 心血管中心 |
| 审查类别 | 初始审查 | 审查时间 | 2024 年 01 月 30 日 |
| 审查方式 | ■会议审查　□紧急会议审查　□快速审查 | | |
| 审查地点 | 姜家园院区 4 号楼 5 楼会议室 | | |
| 审查内容 | 伦理申请审核流程、伦理审查申请表、研究方案、知情同意书样稿 | | |

## 伦理委员会审查决定

■同意　□必要的修改后同意　□必要的修改后重审　□不同意

| | |
|---|---|
| 主任委员/副主任委员签字 | [signature] 南京医科大学第二附属医院医学伦理委员会 |
| 签字日期 | 2024 年 01 月 31 日 |
| 批件有效期 | 2024 年 01 月 31 日~2027 年 01 月 30 日 |
| 伦理委员会 | 南京医科大学第二附属医院医学伦理委员会 |

说明：

1. 研究开展后，本伦理委员会将根据研究实际进展情况，进行跟踪审查。
2. 本伦理委员会联系人：张娟、韩婷，联系电话：025-58509670，邮箱：nydefyll@163.com。
